# Caecal lipoma causing acute appendicitis—a case report

**DOI:** 10.1093/jscr/rjaf030

**Published:** 2025-02-08

**Authors:** Fahreyar Alam, Stewart Chikukuza, Omar Okkeh, Rachel M Jones, Harry R Haynes, Ifrah Omar, Alaa Helal, Filip Tsvetkov

**Affiliations:** General Surgery Department, Great Western Hospital NHS Foundation Marlborough Road, Swindon SN3 6BB, United Kingdom; General Surgery Department, Great Western Hospital NHS Foundation Marlborough Road, Swindon SN3 6BB, United Kingdom; General Surgery Department, Great Western Hospital NHS Foundation Marlborough Road, Swindon SN3 6BB, United Kingdom; General Medicine, Great Western Hospital NHS Foundation Marlborough Road, Swindon SN3 6BB, United Kingdom; Pathology, Great Western Hospital NHS Foundation Marlborough Road, Swindon SN3 6BB, United Kingdom; General Surgery Department, Great Western Hospital NHS Foundation Marlborough Road, Swindon SN3 6BB, United Kingdom; General Surgery Department, Great Western Hospital NHS Foundation Marlborough Road, Swindon SN3 6BB, United Kingdom; General Surgery Department, Great Western Hospital NHS Foundation Marlborough Road, Swindon SN3 6BB, United Kingdom

**Keywords:** caecal lipoma, mesenchymal tumour, appendicitis

## Abstract

Colonic lipoma is a rare mesenchymal tumour of the gastrointestinal tract that is composed of well-differentiated adipose tissue (1). The commonest site of colonic lipomas is the ascending colon (45%), followed by the sigmoid colon (30.3%), descending colon (15.2%), and transverse colon (9.1%) (2). Lipomas of the large intestine represent the third most common benign tumours after hyperplastic and adenomatous polyps (3). They arise from the submucosa in ~90% of cases, but occasionally extend into the muscularis propria; up to 10% are subserosal (3). Colonic lipomas >2 cm may occasionally cause abdominal pain, changes of bowel habits, rectal bleeding, intussusception, bowel obstruction, or prolapse (4). Appendicitis is primarily caused by obstruction of the appendiceal lumen leading to inflammation. In adults, acute appendicitis is commonly attributed to fecaliths, tumours, or infections. In this case we present a patient, who presented with acute appendicitis, secondary to a caecal lipoma.

## Introduction

Colonic lipoma is a rare mesenchymal tumour of the gastrointestinal tract that is composed of well-differentiated adipose tissue [1]. The commonest site of colonic lipomas is the ascending colon (45%), followed by the sigmoid colon (30.3%), descending colon (15.2%), and transverse colon (9.1%) [2]. Lipomas of the large intestine represent the third most common benign tumours after hyperplastic and adenomatous polyps [3]. They arise from the submucosa in ~90% of cases, but occasionally extend into the muscularis propria; up to 10% are subserosal [3]. Colonic lipomas >2 cm may occasionally cause abdominal pain, changes of bowel habits, rectal bleeding, intussusception, bowel obstruction, or prolapse [4].

Appendicitis is primarily caused by obstruction of the appendiceal lumen leading to inflammation. In adults, acute appendicitis is commonly attributed to fecaliths, tumours, or infections.

In this case we present a patient, who was admitted with right iliac fossa pain and mass. He appropriately underwent emergency right hemi colectomy. The histopathology confirmed the diagnosis of a caecal lipoma causing caecal ischemia and obstruction of the appendiceal orifice causing acute appendicitis and severe caecal inflammation.

## Case presentation

A 67-year-old male patient presented to the emergency department with 10 days history of migratory central to right iliac fossa pain, associated with anorexia, nausea, and constipation. There was no history of significant weight loss or any previous surgical intervention. His past medical history included hypertension, chronic kidney disease, IgA nephropathy, Type II diabetes mellites, and obstructive sleep apnoea. His drug history included aspirin, gliclazide, and insulin.

Vital observations were blood pressure (129/63 mmHg), pyrexia (38.2°C), tachycardia of 90/min, SPO2 96%, and respiratory rate of 19/min. General physical examination revealed class III obesity with no pallor, jaundice, or lymphadenopathy. Abdominal examination revealed a slightly distended abdomen with a right iliac fossa mass and localized peritonism. There was no palpable visceromegaly. Hernial orifices were clear and per rectal examination was unremarkable.

Laboratory investigations revealed elevated leukocyte count (14.7 × 10^9^ /L), elevated C-reactive protein level (159 mg/l), and reduced eGFR (27 ml/min).

Initial management included bowl rest, intra venous fluid resuscitation using dextrose-saline, and broad spectrum intra venous antibiotic therapy using Co Amoxiclav (Augmentin 1.2G IV TDS).

**Figure 1:**
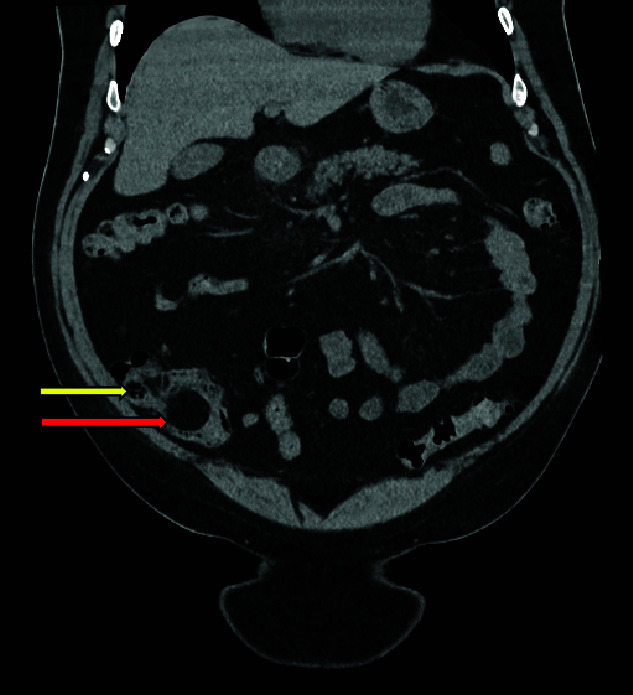
Coronal CT image, showing caecal lipoma (superior arrow) and ileo-caecal valve (inferior arrow).

**Figure 2:**
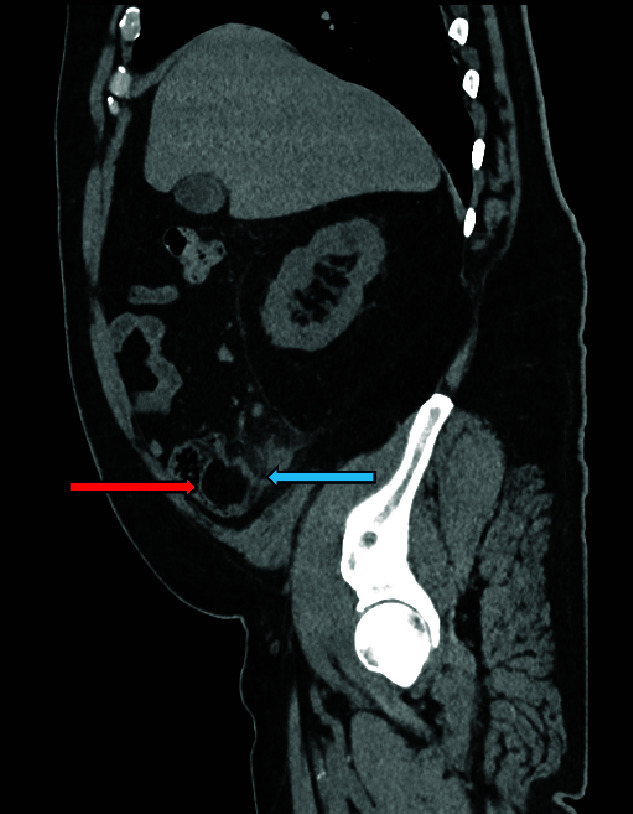
Sagittal CT image, showing caecal lipoma (right arrow) and appendicitis (left arrow).

**Figure 3:**
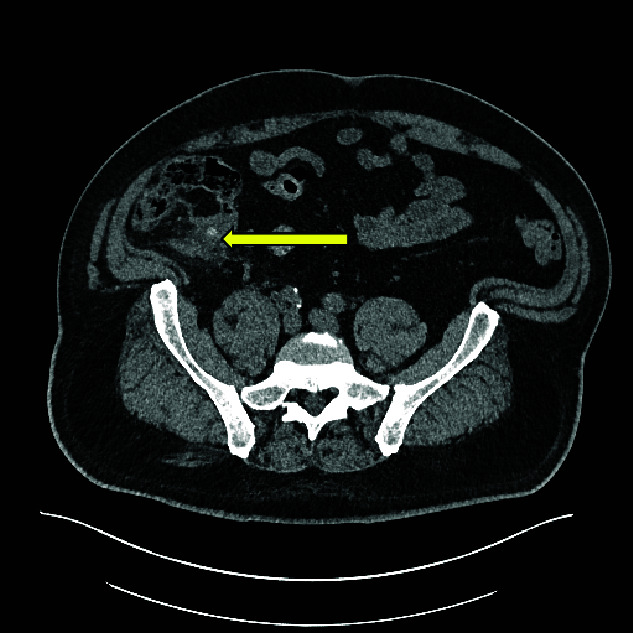
Axial CT image showing caecal inflammation and peri caecal stranding (arrow).

Computed tomography (CT) scan abdomen and pelvis was reported as follows: ‘*Inflammatory phlegmon, fat stranding, free fluid, and reactive lymphadenopathy in the right iliac fossa. At the centre of the inflammatory phlegmon there is a dilated appendix with thickened walls. Small appendicolith at the base. Incidental note of a 3.2 cm caecal lipoma, which resides just below the ileocecal valve*.’ (Figs 1–3).

Considering the clinical presentation, findings, and the CT report, the patient was prepared for emergency surgery.

His P-POSSUM morbidity and mortality was calculated to be 27% and 10.5%, respectively.

Findings at laparoscopy were of an inflammatory mass involving the caecum, proximal ascending colon, terminal ileum, and perforated appendicitis. Decision was taken to convert to a laparotomy. Open right hemi colectomy (side-to-side, double-layered hand-sewn anastomosis was fashioned between the terminal ileum and the mid transverse colon) and thorough washout of the peritoneal cavity was performed.

The patient was transferred to high dependency unit post-operatively. He had an uneventful post-op recovery and was discharged on the 11th post-operative day.

**Figure 4:**
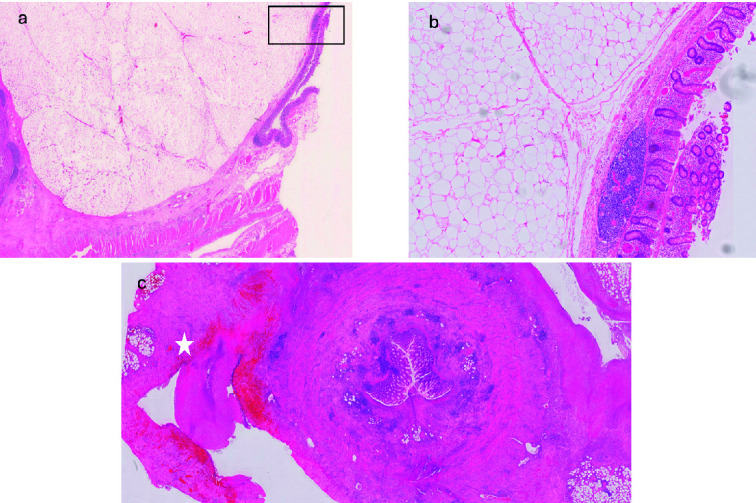
(a) Representative section of the 30 mm mass present at the appendiceal orifice; histology shows a diffuse unencapsulated submucosal mass consisting of sheets of adipocytes; (b) higher power of the area indicated in (a) confirms some mild variation in adipocyte size (likely a degenerate feature) but no evidence of lipoblasts, consistent with a benign submucosal colonic lipoma; (c) appendiceal mucosal ulceration with transmural inflammation, indicating an element of associated acute appendicitis; surrounding the appendix is fibrofatty tissue with extensive acute inflammation and areas of tissue infarction consistent with a peri-appendicular inflammatory mass (star).

Histology showed caecal lipoma at the level of the appendiceal orifice causing caecal ischemia and appendicitis with extensive inflammatory change. No evidence of dysplasia or malignancy was found. Overall, the features were in keeping with caecal ischaemia secondary to a submucosal/mural lipoma with associated appendicitis and a peri-appendicular inflammatory mass (Fig. 4a–c).

## Discussion

Caecal lipoma is an unusual cause of appendicitis. The first report on intestinal lipomatosis focusing on caecal appendix dates back to 1956 in a study by Antoci, and in his study the definite final diagnosis was conducted by histopathologic analysis [5]. Mudd *et al*. [6] reported another case of infarction of a caecal lipoma simulating appendicitis. Hagan *et al*. [7] report a case of acute suppurative appendicitis caused by intussusception of a caecal lipoma involving the appendiceal orifice.

Colonic lipoma was first described by Bauer in 1757. Most colonic lipomas occur in the caecum and ascending colon; they are asymptomatic and need no treatment [8]. Colonic lipomas are often discovered incidentally at colonoscopy, surgery, or autopsy. The most common symptoms are abdominal pain, lower gastrointestinal bleeding, alteration in bowel habits, weight loss, obstipation, intermittent vomiting, etc. Lipomas of the colon may have ulcerated or necrotic overlying mucosa due to chronic pressure effects caused by intussusception, traction, and violent peristalsis, especially if they are pedunculated lipomas [2].

The caecum has a unique anatomical features such as the relative mobility, existence of the appendix and the ileocecal valve, caecal lipoma could have a higher possibility of inducing pathological changes and symptoms [9]. A caecal lipoma occluding the appendicular orifice can cause acute or chronic appendicitis. Tsai *et al.* [9]. reported a case of caecal lipoma with subclinical chronic appendicitis. Chronic appendicitis was thought to be due to partial, but persistent, obstruction of the appendiceal lumen [10].

CT scan is gold standard in recognizing colonic lipomas, presenting with characteristic fatty densitometry values [11]. On CT lipoma has uniform appearance with fat equivalent density in the range of −80 to −120 Hounsfield units and smooth border. The diagnostic value of CT is low for small lipomas [12].

Definitive diagnosis is by histopathology, which shows a tumour consisting of mature fat cells, with a mucous membrane and thin fibrous capsule [13].

Surgical management may involve hemicolectomy, segmental resection, or local excision. Procedure utilized depends on the clinical presentation, imaging and intra operative findings. In our case we started with diagnostic laparoscopy and converted to an open right hemicolectomy.

## Conclusion

Caecal lipoma causing appendicitis is a rare condition associated with locoregional symptoms. Given the rarity of this disease, it is an incidental diagnosis in most cases, following diagnostic imaging for right iliac fossa pain. We have presented a case of a caecal lipoma causing appendicitis that was successfully treated by emergency open right hemicolectomy.

## Data Availability

The authors confirm that the data supporting the findings of this article are available within the article.
